# SCOT: a comparison of cost-effectiveness from a large randomised phase III trial of two durations of adjuvant Oxaliplatin combination chemotherapy for colorectal cancer

**DOI:** 10.1038/s41416-018-0319-z

**Published:** 2018-11-13

**Authors:** José Robles-Zurita, Kathleen A. Boyd, Andrew H. Briggs, Timothy Iveson, Rachel S. Kerr, Mark P. Saunders, Jim Cassidy, Niels Henrik Hollander, Josep Tabernero, Eva Segelov, Bengt Glimelius, Andrea Harkin, Karen Allan, John McQueen, Sarah Pearson, Ashita Waterston, Louise Medley, Charles Wilson, Richard Ellis, Sharadah Essapen, Amandeep S. Dhadda, Rob Hughes, Stephen Falk, Sherif Raouf, Charlotte Rees, Rene K Olesen, David Propper, John Bridgewater, Ashraf Azzabi, David Farrugia, Andrew Webb, David Cunningham, Tamas Hickish, Andrew Weaver, Simon Gollins, Harpreet S Wasan, James Paul

**Affiliations:** 1 Institute of Health and Wellbeing, University of Glasgow, Glasgow, UK; 2 Southampton University Hospital NHS Foundation Trust, Southampton, UK; 3 Department of Oncology, University of Oxford, Oxford, UK; 4 The Christie Hospital, Manchester, UK; 5 Cancer Research UK Clinical Trials Unit, Institute of Cancer Sciences, University of Glasgow, Glasgow, UK; 6 Zealand University Hospital, Department of Oncology and Palliative Care, Rǻdmandsengen 5, DK 4700 Naestved, Denmark; 7 Vall d’Hebron University Hospital and Institute of Oncology (VHIO), Universitat Autònoma de Barcelona, CIBERONC, Barcelona, Spain; 8 Australasian Gastro-Intestinal Trials Group (AGITG) and Monash University and Monash Health, Melbourne, Australia; 9 University of Uppsala, Uppsala, Sweden; 10 Department of Oncology, OCTO, University of Oxford, Oxford, UK; 11 Beatson West of Scotland Cancer Centre, Glasgow, UK; 12 Royal United Hospital, Bath, UK; 13 Addenbrookes Hospital, Cambridge, UK; 14 Royal Cornwall Hospitals NHS Trust, Cornwall, UK; 15 St Luke’s Cancer Centre, Royal Surrey County Hospital NHS Foundation Trust, Guildford, UK; 16 Castle Hill Hospital, Hull, UK; 17 Mount Vernon Cancer Centre, Northwood, UK; 18 Bristol Cancer Institute, Bristol, UK; 19 Barking Havering and Redbridge University Hospital NHS Trust, Barking, UK; 20 Barts Cancer Institute, Queen Mary, University of London, London, UK; 21 University College London, London, UK; 22 Newcastle upon Tyne Hospitals NHS Foundation Trust, Newcastle, UK; 23 Gloucestershire Oncology Centre, Cheltenham General Hospital, Cheltenham, UK; 24 Brighton and Sussex University Hospital Trust, Brighton, UK; 25 Royal Marsden (funded by NIHR BRC at the Royal Marsden), London, UK; 26 Poole Hospital/Bournemouth University, Bournemouth, UK; 27 Churchill Hospital, Oxford University Hospitals Foundation Trust, Oxford, UK; 28 North Wales Cancer Treatment Centre, Rhyl, UK; 29 Hammersmith Hospital, Imperial College London, London, UK

**Keywords:** Health care economics, Economics, Chemotherapy

## Abstract

**BACKGROUND:**

The Short Course Oncology Therapy (SCOT) study is an international, multicentre, non-inferiority randomised controlled trial assessing the efficacy, toxicity, and cost-effectiveness of 3 months (3 M) versus the usually given 6 months (6 M) of adjuvant chemotherapy in colorectal cancer.

**METHODS:**

In total, 6088 patients with fully resected high-risk stage II or stage III colorectal cancer were randomised and followed up for 3–8 years. The within-trial cost-effectiveness analysis from a UK health-care perspective is presented using the resource use data, quality of life (EQ-5D-3L), time on treatment (ToT), disease-free survival after treatment (DFS) and overall survival (OS) data. Quality-adjusted partitioned survival analysis and Kaplan–Meier Sample Average Estimator estimated QALYs and costs. Probabilistic sensitivity and subgroup analysis was undertaken.

**RESULTS:**

The 3 M arm is less costly (-£4881; 95% CI: -£6269; -£3492) and entails (non-significant) QALY gains (0.08; 95% CI: −0.086; 0.230) due to a better significant quality of life. The net monetary benefit was significantly higher in 3 M under a wide range of monetary values of a QALY. The subgroup analysis found similar results for patients in the CAPOX regimen. However, for the FOLFOX regimen, 3 M had lower QALYs than 6 M (not statistically significant).

**CONCLUSIONS:**

Overall, 3 M dominates 6 M with no significant detrimental impact on QALYs. The results provide the economic case that a 3 M treatment strategy should be considered a new standard of care.

## Introduction

Colorectal cancer (CRC) affects 1.36 million patients worldwide each year, and in the UK, is the fourth most common malignancy, accounting for 12% of all new cancer cases each year—~41,265 in 2014.^1^ The cost of treatment for CRC within the first year of diagnosis is considerably larger than that for treating other common cancers such as breast, lung and prostate, and it is estimated that CRC costed the English health-care system £542 million in 2010.^2^ Many authors have also commented on the rising cost of cancer care^3^ and the challenge this presents for high income countries to provide care to increasing populations of cancer patients.

Given recent budget restrictions and rising pressure on health-care systems around the world, there is a need to ensure that cancer treatments offer the best value for money and provide policy makers with up-to-date information on both effectiveness and cost-effectiveness to help inform efficient allocation of health-care resources. In the treatment of CRC, adjuvant chemotherapy is more effective than surgery alone for patients with fully resected CRC at stage III^4–11^ and, to a lesser extent, at high-risk stage II.^12–14^ However, the current 6 month standard duration of adjuvant chemotherapy is associated with considerable side effects^15,16^, which impact on patient quality of life. It has previously been unknown whether 3 months of adjuvant chemotherapy treatment duration could reduce these side effects with no detrimental impact on efficacy. Given the escalating cost of chemotherapy treatments of CRC^17^, it is pertinent that the cost-effectiveness of alternative treatment durations and regimens^18,19^ is explored.

This paper reports on an economic evaluation undertaken alongside the Short Course Oncology Trial (SCOT) randomised controlled trial (ISRCTN No: 23516549) to explore the cost-effectiveness of 3 month (3 M) versus 6 months (6 M) adjuvant chemotherapy treatment in stage III/high-risk stage II CRC patients.

## Methods

The SCOT trial (ISRCTN No.: 23516549) was an international phase III randomised controlled trial, to assess non-inferiority of 3 M versus 6 M of oxaliplatin/FP adjuvant chemotherapy in stage III/high-risk stage II CRC patients. Patients were randomised to receive either 3 or 6 months of oxaliplatin-containing adjuvant treatment. The chemotherapy regimen (FOLFOX or CAPOX) was at the choice of the patient and/or physician prior to randomisation. The trial recruited 6088 patients across six countries (UK, Denmark, Spain, Sweden, Australia and New Zealand), followed up for a minimum of 3 years, up to 8 years post randomisation. SCOT was designed as a non-inferiority trial, aiming to exclude a maximum 2.5% fall in in 3-year disease-free survival from halving the adjuvant treatment duration. Secondary endpoints were overall survival, toxicity, quality of life and cost-effectiveness. Further details of the intervention, randomisation, methods and outcomes for the SCOT trial are reported elsewhere.^20^

The aim of the economic evaluation is to explore the treatment and hospitalisations costs, quality of life and survival outcomes of 3 M versus 6 M adjuvant chemotherapy treatment within the timeframe of the SCOT clinical trial.

The cost-effectiveness analysis was undertaken from the perspective of the UK NHS and Personal Social Services for the 2016 base year, adhering to good practice guidelines.^21,22^ A within-trial analysis utilised the individual patient-level data on resource use, quality of life (EQ-5D-3L) and survival. The cost (C) and quality-adjusted life year (QALY) outcomes for each arm are estimated and combined with the UK decision threshold for cost-effectiveness (λ) of £30,000/QALY^21^ to report outcomes in terms of Net Monetary Benefit (NMB), according to good reporting practice guidance.^23^ (NMB is calculated by monetarising the measure of effectiveness (QALYs) by explicitly incorporating the decision threshold (λ), and then subtracting the cost (C) to determine whether the NMB is greater than zero. Specifically: NMB = QALY × λ –C. The strategy with the greatest NMB is the cost-effective choice.) The incremental NMB is the difference between the NMB of the two arms.

### Outcomes

The effectiveness measure for the economic analysis is the discounted QALY gains per patient in each of the study arms. QALYs are calculated using quality-adjusted survival analysis.^24^ Overall survival is partitioned into three different health states: time on treatment (ToT), disease-free survival after treatment (DFS) and recurrence. The Kaplan–Meier survival estimates are used for the computation of the quality-adjusted survival time in each health state over the 8 year within-trial period.

A separate model estimated quality of life for each health state. EQ-5D-3L data collected for a subsample of 1832 patients (about 30% of the study sample), at baseline and follow-up, and combined with the UK EQ-5D-3L health utility scores^25^ to calculate utilities. A linear regression with standard errors clustered at the individual level estimated quality of life including independent variables: health states, treatment group and individual characteristics.

Time in the health states ToT, DFS and recurrence was computed by integration of the Kaplan–Meier curves and then adjusted by quality of life using the method of integrated quality-survival product^24^ to compute QALYs. This approach to quality-adjusted survival analysis avoids problems of informative censoring in survival analysis based on individual QALYs as an endpoint.^26^

### Costs

Costs were calculated by the measurement and valuation of resources used by the SCOT trial participants during the treatment and follow-up periods. The trial collected patient-level resource use data on adjuvant chemotherapy doses and duration and hospitalisations during treatment and follow-up for the whole study sample. All costs have been valued in 2016 pounds sterling.

Adjuvant chemotherapy: The doses of Oxaliplatin, Capecitabine, 5-fluouracil bolus injection and 5-fluorouracil continuous infusion were collected and combined with their respective unit costs. The cost per mg of each drug is obtained from the British National Formulary 73^27^ as detailed in Table S1 in the supplementary material.

Hospitalisation: Hospitalisation costs are incurred by patients for receiving adjuvant chemotherapy during the treatment period and also for treating adverse reactions. Hospitalisation resource use data include night stays in intensive care unit (ICU), high dependency unit (HDU), general medicine, and in-patient chemotherapy (IP) as well as day cases (DC) and outpatient attendances (OP). The direct and non-direct costs for each hospitalisation were obtained from the Information Services Division (ISD)^28^ of the National Health Service Scotland. Direct costs can be classified by medical, nursing, health professionals, pharmacy, theatre, laboratory and others. For IPs and DCs occurring within the treatment period, the pharmacy cost is subtracted to avoid double counting of chemotherapy medication. Table S2 in the supplementary material details unit costs for each type of hospitalisation. These costs include staff administration time, e.g., for delivering the adjuvant chemotherapy, in medical and nursing costs and non-direct costs when a hospitalisation or appointment for chemotherapy has occurred. The cost of treating adverse events is assumed to be included in hospitalisation costs for patients attending hospital for night, day case or outpatient visit after having adverse reaction.

Kaplan–Meier Sample Average (KMSA) Estimator: Given that the follow-up period differed among patients total cost per patient is estimated by the KMSA estimator.^29^ This way the average total cost is estimated as the sum of the average cost for each period multiplied by the probability of surviving at the beginning of the period.

### Cost-effectiveness outcome

The analyses were performed using STATA 14.0 (StataCorp, TX, USA) to compare the mean costs, and mean QALY differences between the comparator groups (3 months vs. 6 months treatment) and the NMB is reported in line with recent reporting guidelines^23^ and the UK reference case.^21^ Discounting of costs and QALY outcomes beyond 1 year was applied at 3.5% rate as recommended.^21^

### Missing data

Only a subgroup of patients reported EQ-5D health status following recommendations to discontinue data collection based on an interim study analysis.^30^ To control for plausible differences between the EQ-5D sample and the total study sample the quality of life model includes co-variables such as planned treatment, high-risk disease (T4 or N2 as previously defined^20^), gender, age and ethnicity. The model predicts health utilities for the average characteristics of the patients in each health state.

### Sensitivity analysis

Bootstrapping (1000 iterations)^31^ was used to account for uncertainty around the difference in costs and QALYs and how this uncertainty impacts on the cost-effectiveness outcome. The uncertainty is reported through confidence intervals and the computation of cost-effectiveness acceptability probabilities estimated as a function of the threshold for the monetary value of a QALY.^32^

Subgroup analyses was undertaken, in line with the main trial analysis,^20^ to consider cost-effectiveness of the two treatment duration strategies according to: planned treatment regimen, CAPOX/FOLFOX; disease risk, high/low risk stage III; gender; and age.

## Results

### Health outcomes

**Fig. 1:**
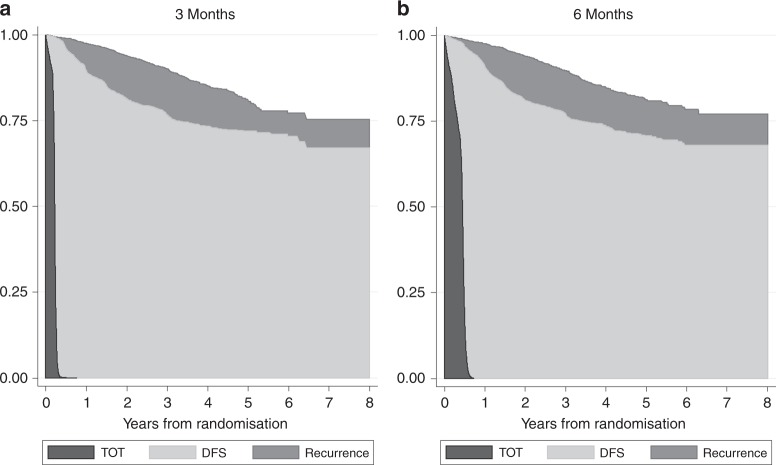
Overall survival partitioned into time on treatment (ToT), disease free survival after treatment (DFS), and recurrence. Kaplan-Meier estimates over 8 years and by arms

In total, 6088 patients were randomised in the SCOT study, however, only 6065 patients are considered in the analysis since 23 patients withdrew consent to use their data at follow-up.^20^ Table 1 illustrates how overall expected survival time, over 8 years after randomisation, is split into ToT, DFS and recurrence. ToT is significantly higher in the 6 M arm while DFS just fails to significantly favour the 3 M arm at 5% of error. No statistically significant differences are found for time in recurrence or overall survival. Figure 1 shows the area representing ToT, DFS and recurrence time generated from Kaplan–Meier estimates (see more detailed Kaplan–Meier curves in supplementary material: Figures S1, S2 and S3).

**Table 1:** Overall survival time by health state (ToT, DFS and recurrence) and by arm

	3 M	6 M	Incremental	
Survival analysis	Mean	Mean	Mean	*P*-value
*N*	3035	3030	6065	
ToT	0.21	0.39	−0.18	0.000
DFS	5.93	5.74	0.19	0.053
Recurrence	0.73	0.77	−0.041	0.605
Total (OS)	6.87	6.90	−0.032	0.695

Kaplan–Meier estimates used for computation of expected time in each health state

Survival time estimated up to 8 years post randomisation

Estimation sample in the case of ToT is lower due to missing values, 3018 and 3013 patients for the 3 M and 6 M arms, respectively

### Quality of life

**Fig. 2:**
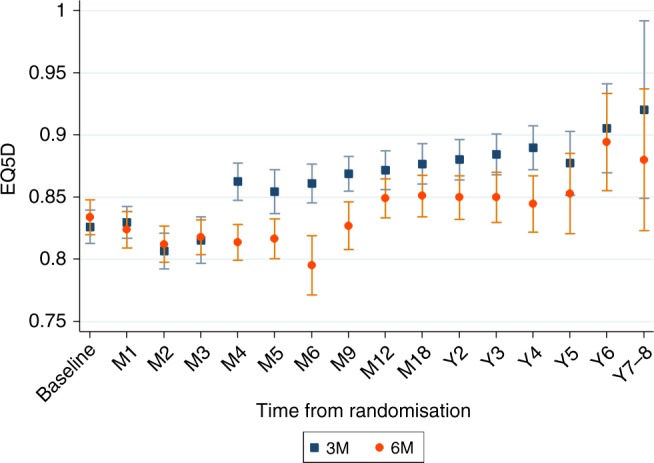
Evolution of EQ-5D utilities over time by arms. Average and 95% CI

Table 2 shows the results of the utility model for non-missing observations. The effect of recurrence and time on treatment is capture by two indicators taking value 1 for all those EQ-5D responses occurring in those health states. ToT and recurrence have a significant negative effect on utility, as could be expected. The 3 M arm is estimated to have higher quality of life (*P*-value < 0.05) even after controlling for recurrence and time on treatment. These results are consistent with the higher incidence of adverse events in the 6 M groups compared with the 3 M arm. For example, the main long-term adverse event was neuropathy associated with the Oxaliplatin component of the chemotherapy regimen. Specifically, 58% of patients in the 6 M arm had peripheral neuropathy equal or worse than Grade 2, the same figure for the 3 M arm was 25%. Patients’ characteristics are included in the model to adjust health utilities to the average values of the whole SCOT sample, with and without EQ-5D data (see Table S3 in supplementary material for differences in characteristics between subsamples). Significant characteristics are *male* and *age*, with a positive effect, and ethnicity, with a lower quality of life for African/Caribbean patients and South Asian with respect to White/Caucasian. No significant variables are planned treatment and disease risk. The results of the model follow the pattern of the evolution of EQ-5D scores over time for both arms in Fig. 2. Figures S1–S5 From randomisation point quality of life decreases for both arms up to 3 months. At this point health utilities for those in 3 M arm increases as they finish treatment while those patients in the 6 M arm continue with lower quality of life. Changes in quality of life are related to time on treatment although differences between the two arms remain after receiving adjuvant chemotherapy to some extent.

**Table 2:** Health utilities regression

Variable	Coef.	S.E.
N-observations	16,091	
N-patients	1757	
Health states (ref: disease free)		
On treatment	−0.0394^***^	0.00408
Recurrence	−0.0578^***^	0.0139
Arm: 6 months	−0.0154^*^	0.00730
Characteristics		
CAPOX	0.00402	0.00783
High risk	−0.00911	0.00724
Male	0.0159^*^	0.00733
Age	0.00162^***^	0.000429
Ethnic (ref: White/Caucasian)		
African/Caribbean	−0.0810^*^	0.0385
South Asian	−0.145^**^	0.0536
Chinese	−0.0447	0.0772
Other	0.0178	0.0217
Constant	0.866^***^	0.00944

Standard errors (S.E.) clustered at the patient level

*P*-values: ^*^P < 0.05, ^**^P < 0.01^, ***^P < 0.001

N-observations refer to the number of EQ-5D questionnaires reported in total by the N-patients included in the estimation

The constant in the model refers to a 65-year-old female patient, in disease-free health state in the 3 months arm, receiving FOLFOX treatment, with low risk stage III and White/Caucasian ethnicity

### Costs

Table 3 details cost incurred by patients in both arms, with a breakdown of the hospitalisation costs over different time periods. As expected, adjuvant chemotherapy costs are higher in the 6 M arm. Hospitalisation costs differ between arms in the 4–6-month period, as could be expected; however, the data also reveal that patients in the 6 M arm experience more hospitalisation costs in the 7–12-month period, possibly reflecting longer lasting complications due to the longer treatment period. Interestingly, the costs during the first 6 months after randomisation are not doubled for the 6 M arm. Specifically, adjuvant chemotherapy and hospitalisation costs are only 1.67 and 1.45 times higher, respectively, in the 6 M arm. This is due to tolerability of treatment where many patients randomised to 6 M do not complete the full course of treatment. While 83.3% of patients randomised to 3 M received the full 3 months treatment, only 58.8% of those randomised to 6 M received 6 months treatment.^20^ Notice that the health economic evaluation is clearly justified in the context of this study to analyse the actual consequences on costs from reducing the intended treatment duration from 6 to 3 months. There is no difference in cost between arms beyond 12 months. Overall, the cost is significantly greater in the 6 M arm, driven by hospitalisations (-£2835) in the first 6 months to a higher extent than by received adjuvant chemotherapy (-£1829). A detailed analysis of resource use, regarding adjuvant chemotherapy and hospitalisations, can be found in Tables S4 and S5, respectively, in the supplementary material.

**Table 3:** Costs by treatment duration (£/patient)

Time from beginning of treatment	3 M	6 M	Incremental	
	Mean	Mean	Mean	*P*-value
*N*	3035	3030	6065	
Adjuvant chemotherapy	2750	4579	−1829	< 0.001
Hospitalisation (0–3 months)	3576	3595	−19	0.816
Hospitalisation (4–6 months)	1790	4185	−2395	< 0.001
Hospitalisation (7–12 months)	2748	3054	−306	0.030
Hospitalisation ( > 12 months)	8473	8588	−115	0.876
Total hospitalisation	16587	19422	−2835	< 0.001
Total	19337	24001	−4663	< 0.001

Figures refer to non-discounted average cost for each period conditional on survival

### Cost-effectiveness analysis

Table 4 details the results of the base case cost-effectiveness analysis. The 3-month adjuvant chemotherapy strategy is significantly cheaper, costing £4,881 less than the 6 M strategy over the 8 year analysis period. The 3 M strategy also results in the greatest QALY gain expected, although this is not statistically significant (*P*-value = 0.33) and results in uncertainty in the QALY gains as seen in the 95% confidence intervals. The QALY gains for the 3 M arm are driven by the significant improvement in quality of life rather than life expectancy (LE), indeed the 6 M arm has a very small non-significant improvement in LE (*P*-value = 0.69), meeting the non-inferiority margin for survival specified for the SCOT trial.^20^ These cost-effectiveness results indicate that the 3 M treatment strategy is dominant (cost saving with improvement in QALYs), with an incremental NMB of £7246 per patient. 3 M strategy is therefore the cost-effective option, with 99% probability of being cost-effective at the UK decision threshold of £30,000/QALY.^21^ The 3 M also remains the optimal choice across a wide range of willingness to pay values (see Figure S4 in supplementary material for the cost-effectiveness acceptability curve).

**Table 4:** Estimates of costs and QALYs for the two strategies

Intervention strategies	Costs (£/patient)		LE		QALYs		NMB (£30 K/QALY)		Prob CE λ = £30,000
	Mean	[95% CI]	Mean	[95% CI]	Mean	[95% CI]	Mean	[95% CI]	
3 Months	18,401	[17,538; 19,328]	6.87	[6.73; 6.99]	5.30	[5.17; 5.40]	140492	[135,327; 145,658]	0.995
6 Months	23282	[22,227; 24,367]	6.90	[6.78; 7.02]	5.22	[5.10; 5.34]	133246	[129,569; 136,922]	0.005
Incremental	−4881	[−6269; −3492]	−0.03	[−0.22; 0.13]	.08	[−0.086; 0.230]	7246	[3469; 11,023]	(3 M dominates)

Health utilities conditional on survival considered

KMSA estimator and partitioned survival analysis used for costs and QALYs, respectively

CIs are computed using bootstrap sampling

Probability of cost-effectiveness calculated using 1000 bootstrap replications

### Subgroup analysis

Table S6 illustrates the cost-effectiveness for the subgroup analyses according to: (i) treatment regimen, (ii) risk, (iii) gender and (iv) age. For all the subgroups except FOLFOX treatment and males, the cost-effectiveness results are similar to the base case analysis with the 3 M arm dominating and a 99% probability of being the cost-effective option. In the FOLFOX treatment group, the 3 M arm shows cost saving, but also fewer QALY gains, which were not significant but driven by a small gain in LE for the 6 M arm. The relative advantage of the 3 M arm in QALYs is higher for the CAPOX treatment, high-risk patients, females and older patients. An interaction test of differences in incremental QALYs between subgroups conclude that only the treatment regimen subgroup shows a statistically significant difference in QALY differences, CAPOX (0.19 Incremental QALYs) and FOLFOX (−0.12 Incremental QALYs) with a *P*-value = 0.066. This relative advantage of the 6 M for the FOLFOX subgroup is driven by life expectancy (see Table S7 in supplementary material for a detailed subgroup survival analysis). While for the CAPOX regimen, life expectancy is better for the 3 M arm by 0.07 years, patients treated with FOLFOX have a greater life expectancy in the 6 M arm, by 0.22 years (interaction test, *P*-value = 0.106). In the FOLFOX subgroup, the 3 M arm still maintains a greater NMB than 6 M, with an incremental NMB of £3229 and a 77% probability of being cost-effective given a UK threshold of £30,000/QALY. Therefore, the 6 M arm would not be considered cost-effective and the 3 M treatment duration remains the optimal treatment strategy.

The interaction test of differences in incremental QALYs between the risk, gender and age subgroups showed no significant differences in incremental QALYs.

For each subgroup, the 3 M strategy has the highest probability of being cost effective for a wide range of monetary values of a QALY (see Figure S5 in supplementary material for the cost-effectiveness acceptability curves). Only when a threshold higher than £60,000/QALY is considered the 6 M arm becomes the cost-effective strategy (with highest probability) for the FOLFOX subgroup.

## Discussion

The results of the economic evaluation show that the 3-month duration arm is clearly a cheaper intervention and the dominant strategy for chemotherapy treatment for high-risk stage II and stage III CRC. A short treatment duration significantly reduces costs by saving resources related to adjuvant chemotherapy and hospitalisations. The 3 M arm also significantly improves patient quality of life during the treatment period, with no significant impact on overall survival, leading to an overall QALY gain for the 3 M arm, albeit not statistically significant. The probabilistic analysis and exploration of cost-effectiveness acceptability showed little uncertainty in the economic results over a wide range of willingness to pay thresholds.

An exploration of four subgroup analyses indicates similar conclusions, with the 3 M arm being the dominant or cost-effective strategy given the UK £30,000 per QALY threshold. The type of chemotherapy received has the greatest subgroup impact on cost-effectiveness. The interaction between planned treatment and QALY differences between the two arms is statistically significant. In the analyses of the FOLFOX chemotherapy regimen, the 6 M arm generated more QALYs than the 3 M arm, primarily driven by the higher estimated disease free and overall survival, which was explored and discussed in the main study analyses.^20^ The quality of life difference between arms is in favour of the 3 month arm, yet the survival gains in the FOLFOX regimen are relevant over the 8 year follow-up period, resulting in an overall QALY gain. Nonetheless, at a decision threshold of £30,000/QALY the NMB is larger for the 3 M arm (incremental gain of £3229 per patient) and therefore the 6 M duration would not be considered cost-effective from a UK perspective.^21^

Strengths: The 8 year patient follow-up allowed exploration of the cost-effectiveness outcomes over this long time period. The key cost differences occur within the first year after randomisation, where the 6 M arm costs nearly double the 3 M arm in chemotherapy and hospitalisations. Between years 1 to 8 there is little difference in hospitalisations between arms, yet over the 8-year analysis period the total costs per patient are significantly different between arms, favouring the 3 M arm. We would not expect longer-term differences in costs between arms. The follow-up also allows a thorough consideration of the QALY results which are driven by the timing of events. There are small differences in life expectancy which are subject to uncertainty in both the base case and subgroup analyses, yet, overall quality of life is consistently higher in the 3 M arm in all analyses. The maximum advantage in quality of life is between 3 to 6 months post randomisation where the 3 M arm have stopped treatment. The detrimental quality of life impact on the 6 M arm remains due to the adverse event derived from chemotherapy.^30^

Limitations: Data present censoring given the minimum of 3-year follow up and the relatively high survival rates after 8 years (about 75%). This aspect could be affecting the results to some extent. The cost and quality-adjusted survival methodology used, i.e., KMSA and partitioned survival analysis respectively, is chosen to reduce censoring-related biases.

This study was undertaken using the within-trial 8 years time horizon. The analysis shows that the key differences between arms in terms of costs and quality of life occur mainly within the first year, and beyond this point there are no significant differences between the two strategies. Nonetheless, extrapolation beyond the 8 years is unlikely to change the outcomes and conclusions unless we anticipate huge differences in survival.

## Conclusion

This study found that compared with the traditional 6-month chemotherapy treatment period, a 3 months treatment strategy costs significantly less with no significant detrimental impact on patient outcomes (quality of life and survival) and therefore is found to dominate. Three months chemotherapy treatment for patients with stage III and high-risk stage II CRC is the optimal treatment strategy from an UK health-care perspective. Cost-effectiveness is affected by the type of chemotherapy regimen used, however, the 3 M strategy remains the optimal choice for both the CAPOX and FOLFOX regimens under a policy relevant willingness to pay thresholds.

The SCOT trial was the largest randomised study in adjuvant treatment of CRC to date, which showed that 3 M treatment is non-inferior to 6 M treatment in the overall trial population. This economic evaluation undertaken alongside SCOT adds to the evidence showing that the 3 M treatment strategy is not only cost-effective, but a dominant treatment strategy with little uncertainty in the cost-effectiveness decision; thus providing the economic case that a 3 M treatment strategy should be considered a new standard of care.

## Electronic supplementary material

Supplementary material

Supplementary material

Supplementary material

Supplementary material

Supplementary material

Supplementary material
