# Supplementary figures and images for: SCOT: a comparison of cost-effectiveness from a large randomised phase III trial of two durations of adjuvant Oxaliplatin combination chemotherapy for colorectal cancer
Source: Br J Cancer. 2018 Nov 13;119(11):1332–8. doi: 10.1038/s41416-018-0319-z (PMC6265336; doi:10.1038/s41416-018-0319-z)

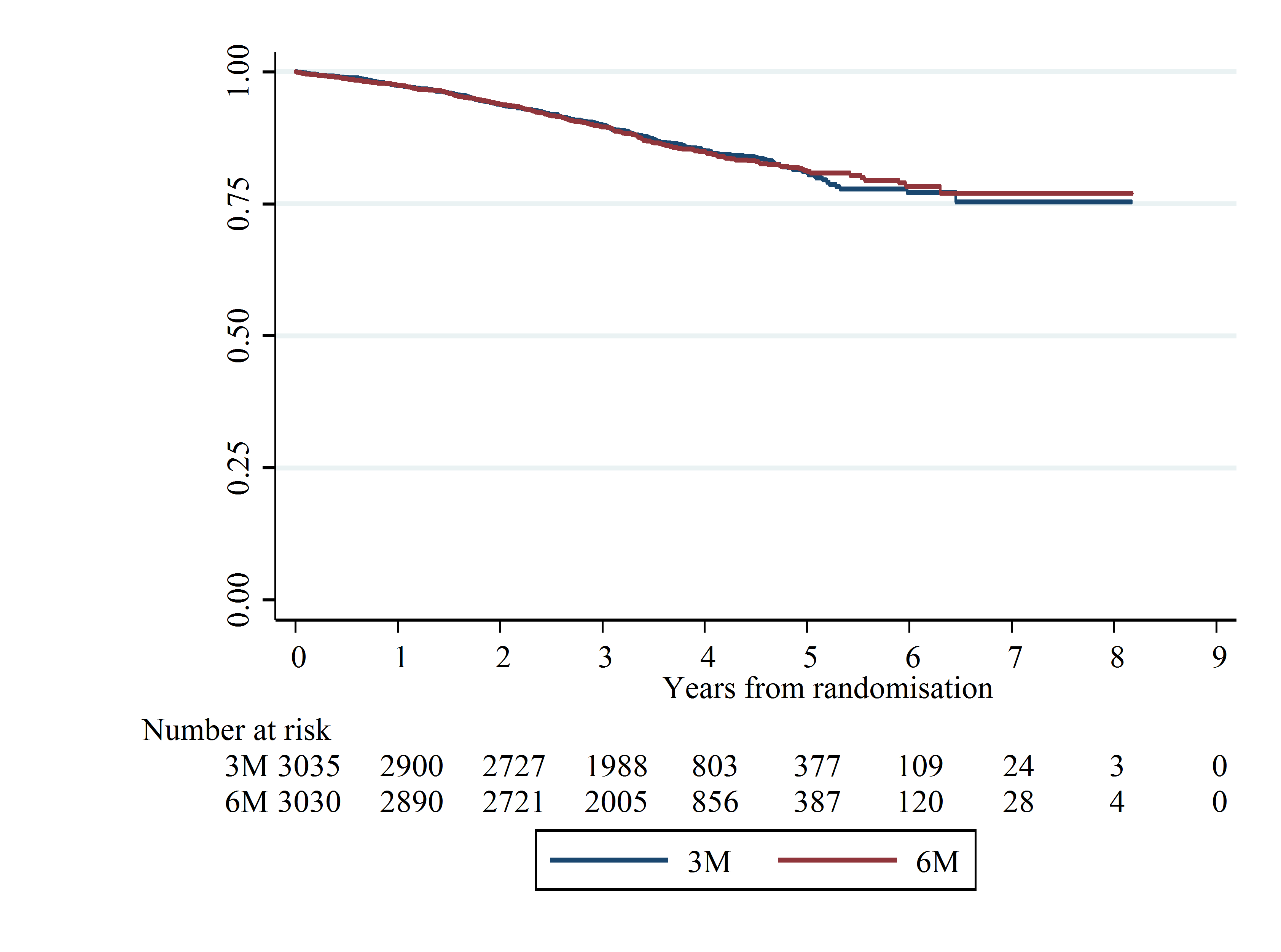

Supplement: Supplementary file 1 — Supplementary material [file 41416_2018_319_MOESM1_ESM.tif]

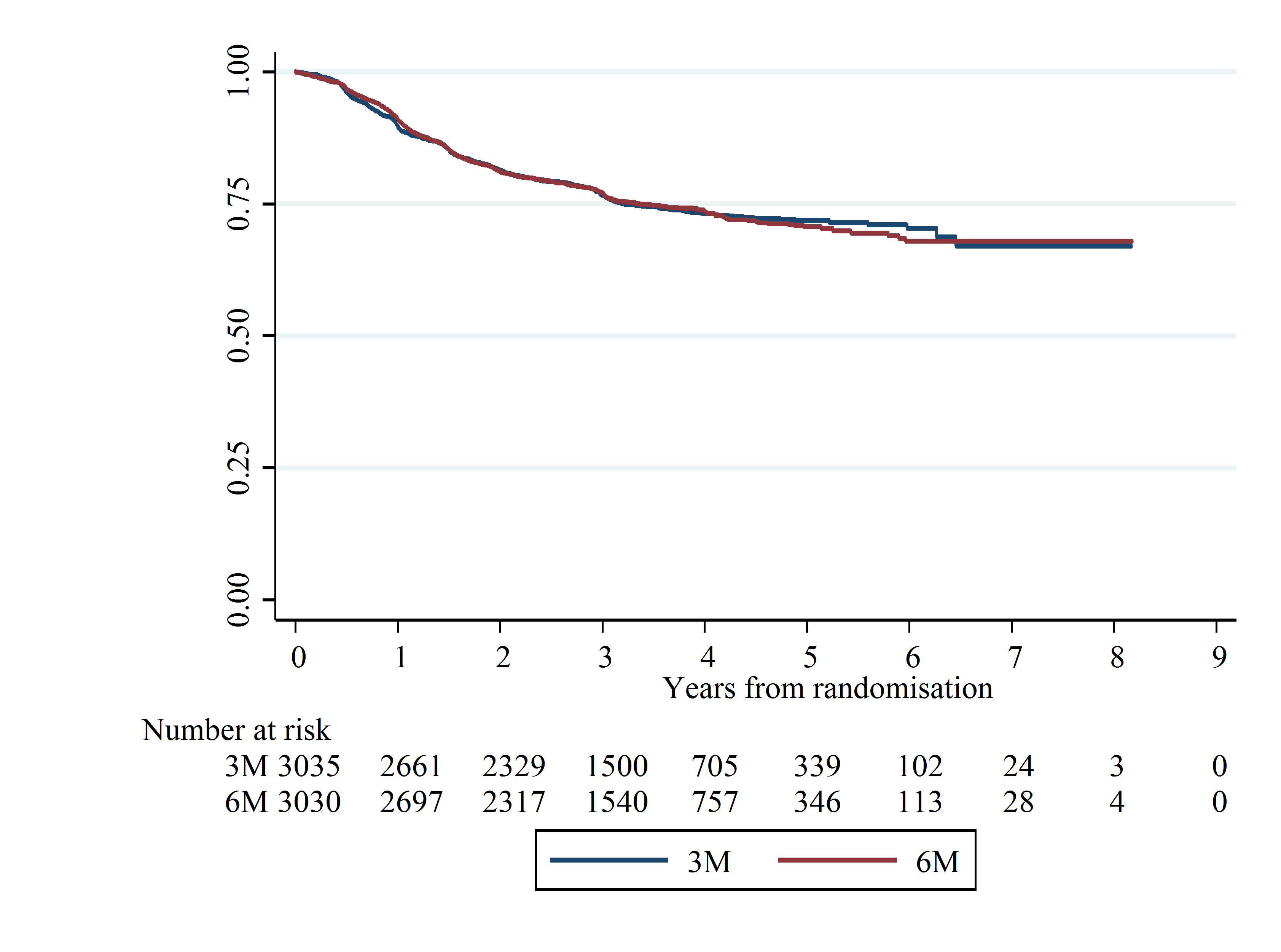

Supplement: Supplementary file 2 — Supplementary material [file 41416_2018_319_MOESM2_ESM.tif]

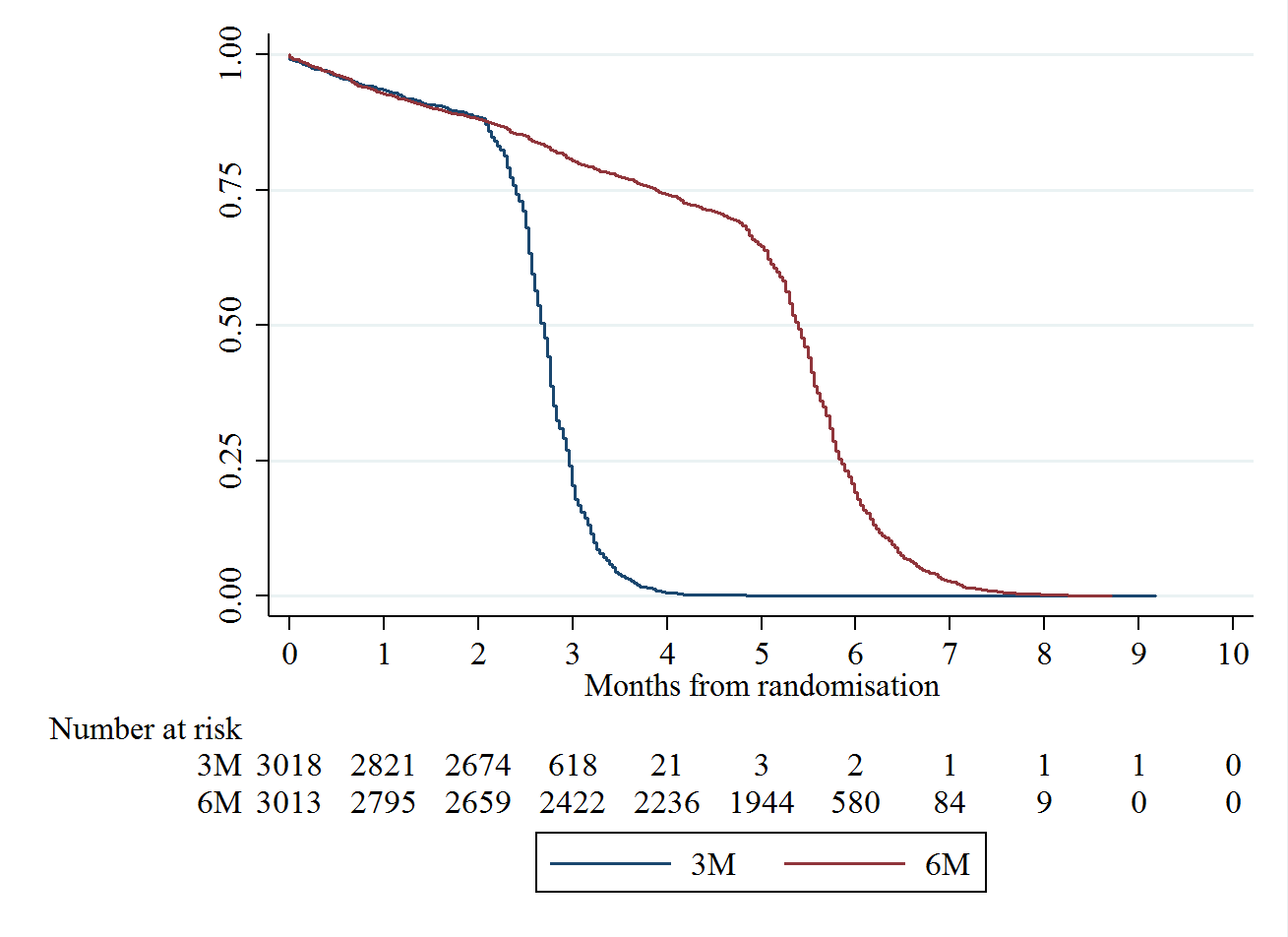

Supplement: Supplementary file 3 — Supplementary material [file 41416_2018_319_MOESM3_ESM.tif]

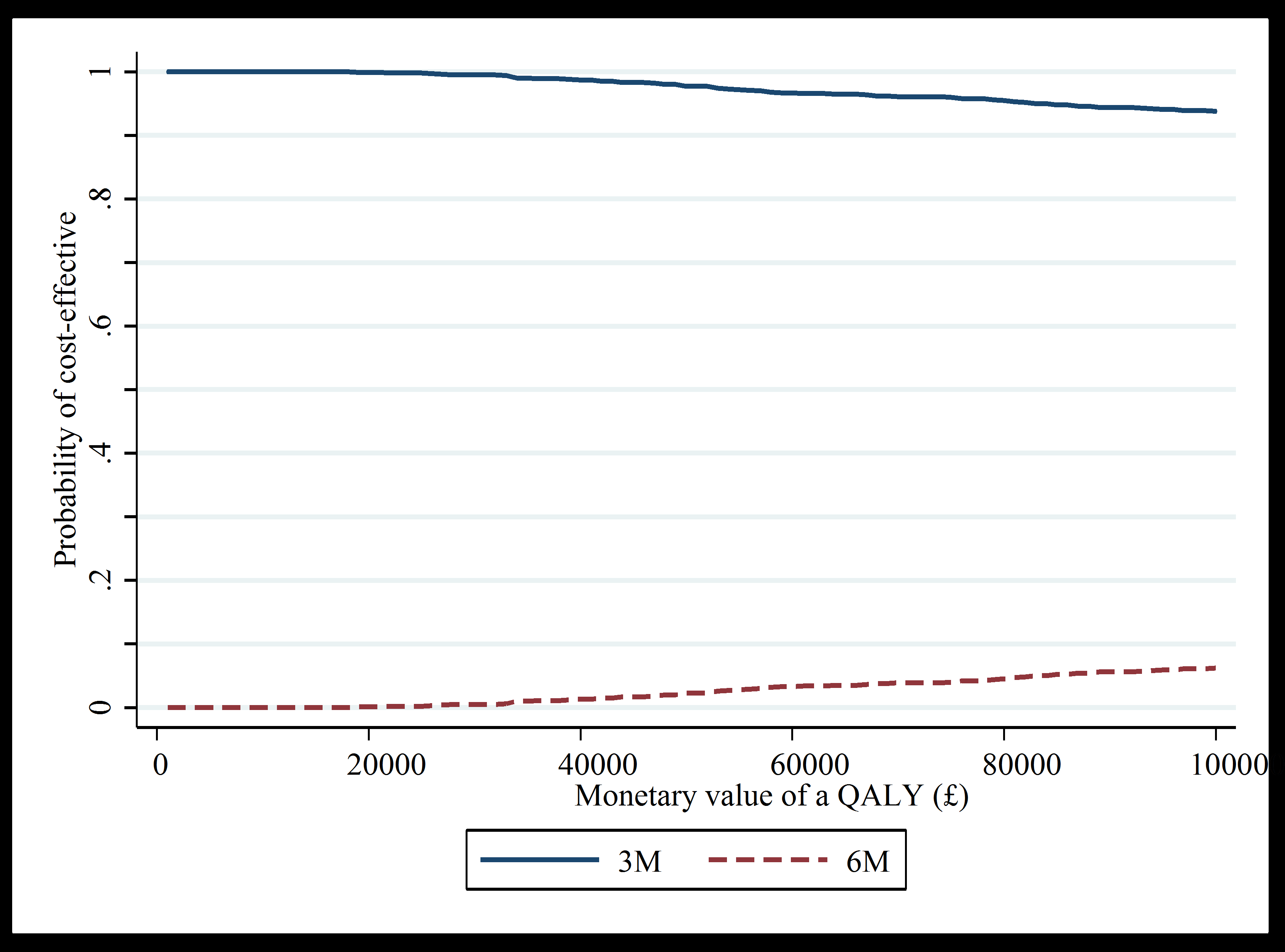

Supplement: Supplementary file 4 — Supplementary material [file 41416_2018_319_MOESM4_ESM.tif]

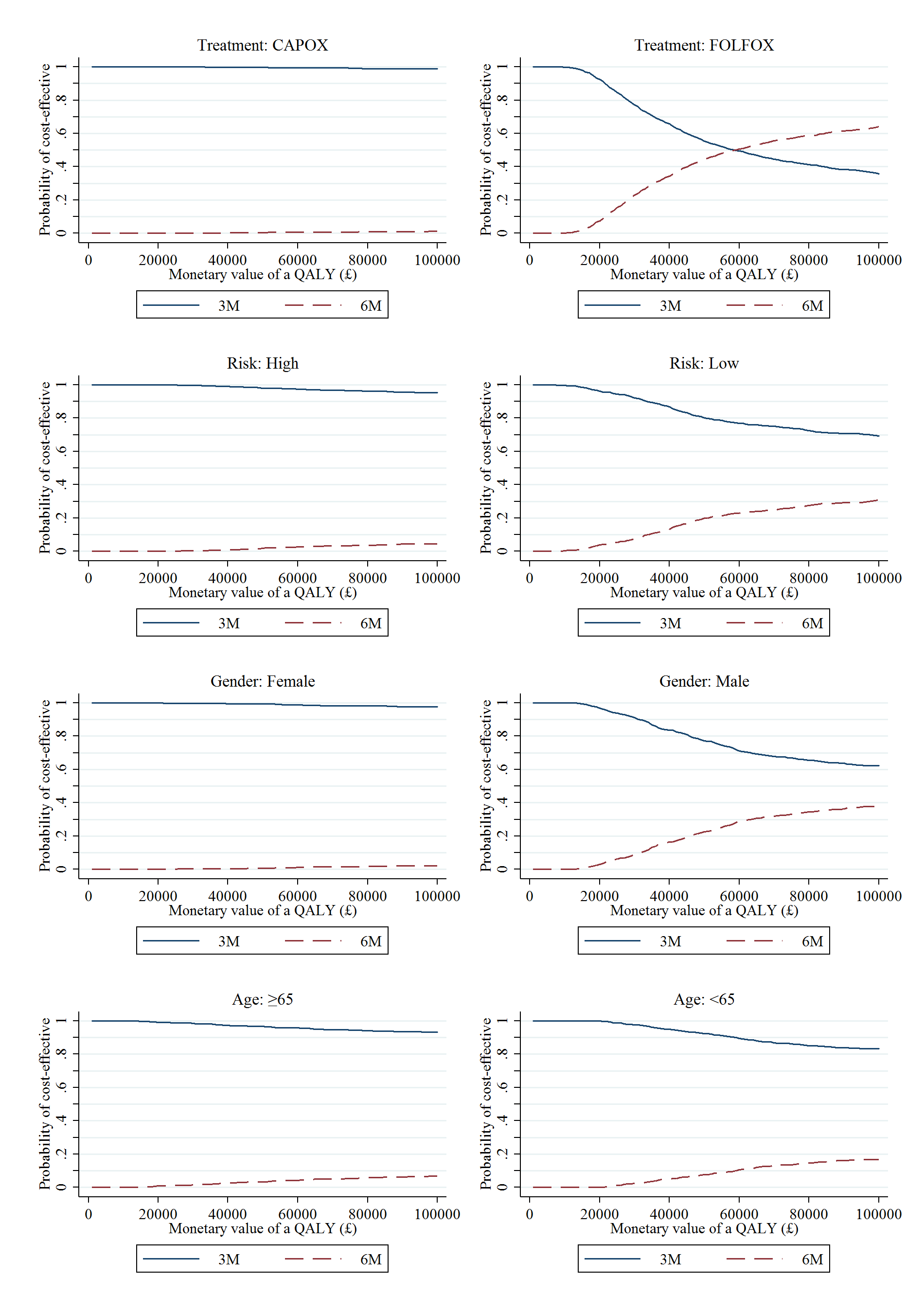

Supplement: Supplementary file 5 — Supplementary material [file 41416_2018_319_MOESM5_ESM.tif]
