# Supplementary material for: SCOT: a comparison of cost-effectiveness from a large randomised phase III trial of two durations of adjuvant Oxaliplatin combination chemotherapy for colorectal cancer
Source: Br J Cancer. 2018 Nov 13;119(11):1332–8. doi: 10.1038/s41416-018-0319-z (PMC6265336; doi:10.1038/s41416-018-0319-z)
Supplement: Supplementary file 6 — Supplementary material [file 41416_2018_319_MOESM6_ESM.docx]

**Tables in supplementary material**

Supplementary 1. Unit costs

| Table S1. Unit costs. Adjuvant chemotherapy | | | |
| --- | --- | --- | --- |
|  | | | |
| Drug | Description | Cost (£) | Unit cost (£/mg) |
| Oxaliplatin | Oxaliplatin 200mg/40ml concentrate for solution for infusion vials | 595.65 | 2.98 |
| Capecitabine | Capecitabine 500mg tablets 120 tablets | 146 | $2.43\times{10}^{-3}$ |
| 5-fluoracil (bolus) | Fluorouracil 500mg/20ml solution for injection vials 10 vial | 64 | $1.28\times{10}^{-2}$ |
| 5-fluoracil (infusion) | Fluorouracil 2.5g/100ml solution for infusion vials | 32 | $1.28\times{10}^{-2}$ |
| Note 1. Chemotherapy drugs described as by the British National Formulary (BNF) 2017  Note 2. The lowest price medication for each drug at the appropriate dosage was used, BNF 2017 | | | |

| Table S2. Unit costs. Hospitalisation | | | | | | | | | |
| --- | --- | --- | --- | --- | --- | --- | --- | --- | --- |
| Hospitalisations^*^ | Cost per night or case (£) | | | | | | | | |
|  | Medical | Nursing | AHP | Pharmacy | Theatre | Laboratory | Other | Non direct costs | Total cost |
| ICU^+^ | 284.24 | 1029.79 | 36.63 | 263.48 | 9.83 | 76.44 | 22.35 | 467.59 | 2,190.35 |
| HDU^+^ | 125.74 | 423.47 | 13.82 | 87.07 |  | 41.43 | 6.40 | 239.95 | 937.87 |
| General medicine^+^ | 73.12 | 161.03 | 16.60 | 47.80 | 3.77 | 25.77 | 5.81 | 142.82 | 476.73 |
| In-patient (clinical oncology) ^+^ | 103.47 | 198.66 | 74.87 | 157.15 |  | 30.71 | 38.25 | 293.77 | 896.88 |
| Out-patient (clinical oncology) ^++^ | - | - | - | - | - | - | 192.40^+^ | 59.37 | 251.77 |
| Day case (clinical oncology) ^+++^ | 147.27 | 77.37 | 51.67 | 281.32 |  | 17.41 | 24.84 | 213.34 | 813.22 |
| Note 1. ICU - Night at intensive care unit; HDU - Night at high dependency unit; General medicine - Night at general medicine unit; In-patient (clinical oncology) - Night at clinical oncology as in-patient; Outpatient (clinical oncology) - Out-patient attendance for clinical oncology; Day case (clinical oncology) - Day case at clinical oncology.  Note 2. Source of information: * Information Services Division, Scotland 2017; + ISD report R040; ++ ISD report R044; +++ ISD report R042.  Note 3. All indirect cost for *Outpatient* are included in the *Other* category. | | | | | | | | | |

Supplementary 2. Distribution of characteristics of the EQ5D vs. non-EQ5D samples

| Table S3. Characteristics of patients by EQ5D subsample | | | | |
| --- | --- | --- | --- | --- |
|  | Total | Non-EQ5D | EQ5D | P-value |
| *N* | 6,065 | 4,308 | 1,757 |  |
| Planned treatment |  |  |  |  |
| CAPOX | 67.5 | 67.5 | 67.5 |  |
| FOLFOX | 32.5 | 32.5 | 32.5 | 0.99 |
| Gender |  |  |  |  |
| Female | 39.46 | 39.65 | 38.99 |  |
| Male | 60.54 | 60.35 | 61.01 | 0.633 |
| Age | 63.43 | 63.35 | 63.62 | 0.3094 |
| Disease risk |  |  |  |  |
| High | 53.19 | 54.32 | 50.43 |  |
| Low | 46.81 | 45.68 | 49.57 | 0.006 |
| Ethnicity |  |  |  |  |
| White/Caucasian | 94.02 | 82.61 | 77.95 |  |
| African/Caribbean | 1.37 | 1.09 | 0.97 |  |
| South Asian | 1.42 | 1.02 | 0.86 |  |
| Chinese | 0.4 | 0.25 | 0.19 |  |
| Other | 2.79 | 15.04 | 20.03 | <0.001 |
| Note. Differences between groups tested with Chi2-test (planned treatment, gender, disease risk, and ethnicity) or t-test (age). | | | | |

Supplementary 3. Resources use

| Table S4. Adjuvant Chemotherapy received by intervention and planned regimen (mg/patient) | | | | | | | | | | | | |
| --- | --- | --- | --- | --- | --- | --- | --- | --- | --- | --- | --- | --- |
|  | All patients | | | | FOLFOX | | | | CAPOX | | | |
|  | 3M | 6M | Incremental | | 3M | 6M | Incremental | | 3M | 6M | Incremental | |
| Type of drug | Mean | Mean | Mean | P-value | Mean | Mean | Mean | P-value | Mean | Mean | Mean | P-value |
|  |  |  |  |  |  |  |  |  |  |  |  |  |
| OX | 842.1 | 1,273.0 | -430.8 | <0.001 | 875.8 | 1,352.5 | -476.7 | <0.001 | 826.0 | 1,234.5 | -408.5 | <0.001 |
| CAP | 114,142.1 | 192,930.5 | -78,788.4 | <0.001 | 3,622.8 | 7,110.4 | -3,487.7 | 0.031 | 167,165.6 | 282,702.6 | -115,537.0 | <0.001 |
| T5FUB | 1,293.9 | 2,184.6 | -890.7 | <0.001 | 3,871.6 | 6,391.7 | -2,520.1 | <0.001 | 57.3 | 152.1 | -94.8 | <0.001 |
| T5FUIV | 8,012.8 | 14,196.1 | -6,183.3 | <0.001 | 23,991.7 | 41,645.9 | -17,654.3 | <0.001 | 346.6 | 934.7 | -588.1 | <0.001 |
| *N* | 3,035 | 3,030 | 6,065 |  | 984 | 987 | 1,971 |  | 2,051 | 2,043 | 4,094 |  |
| Note. OX – Oxaliplatin, CAP – Capecitabine, T5FUB - 5-fluorouracil bolus injection, T5FUIV – 5-fluorouracil continuous infusion. | | | | | | | | | | | | |

| Table S5. Hospital resources by intervention and planned regimen (night, days or appointment per patient) | | | | | | | | | | | |  |
| --- | --- | --- | --- | --- | --- | --- | --- | --- | --- | --- | --- | --- |
|  | All patients | | | | FOLFOX | | | | CAPOX | | |  |
|  | 3M | 6M | Incremental | | 3M | 6M | Incremental | | 3M | 6M | Incremental |  |
|  | Mean | Mean | Mean | P-value | Mean | Mean | Mean | P-value | Mean | Mean | Mean | P-value |
| 0-3 months |  |  |  |  |  |  |  |  |  |  |  |  |
| ICU | 0.013 | 0.022 | -0.010 | 0.222 | 0.006 | 0.020 | -0.014 | 0.198 | 0.016 | 0.023 | -0.007 | 0.475 |
| HDU | 0.017 | 0.029 | -0.012 | 0.391 | 0.008 | 0.014 | -0.006 | 0.520 | 0.022 | 0.037 | -0.015 | 0.462 |
| GEN | 0.846 | 0.753 | 0.093 | 0.230 | 0.758 | 0.661 | 0.098 | 0.421 | 0.888 | 0.798 | 0.090 | 0.361 |
| IP | 0.143 | 0.148 | -0.005 | 0.859 | 0.186 | 0.209 | -0.023 | 0.669 | 0.122 | 0.118 | 0.004 | 0.873 |
| OP | 4.588 | 4.633 | -0.045 | 0.667 | 6.777 | 6.522 | 0.256 | 0.274 | 3.538 | 3.721 | -0.183 | 0.045 |
| DC | 2.643 | 2.653 | -0.009 | 0.919 | 4.254 | 4.193 | 0.062 | 0.784 | 1.871 | 1.909 | -0.038 | 0.583 |
| 3-6 months |  |  |  |  |  |  |  |  |  |  |  |  |
| ICU | 0.042 | 0.012 | 0.030 | 0.119 | 0.013 | 0.018 | -0.005 | 0.743 | 0.055 | 0.009 | 0.046 | 0.088 |
| HDU | 0.011 | 0.009 | 0.002 | 0.785 | 0.006 | 0.017 | -0.011 | 0.459 | 0.013 | 0.005 | 0.008 | 0.293 |
| GEN | 0.524 | 0.203 | 0.321 | 0.000 | 0.611 | 0.226 | 0.385 | 0.005 | 0.482 | 0.191 | 0.291 | 0.001 |
| IP | 0.036 | 0.081 | -0.045 | 0.005 | 0.029 | 0.128 | -0.098 | 0.002 | 0.039 | 0.058 | -0.020 | 0.282 |
| OP | 2.123 | 3.585 | -1.462 | 0.000 | 2.408 | 5.200 | -2.792 | 0.000 | 1.987 | 2.805 | -0.818 | 0.000 |
| DC | 0.272 | 1.980 | -1.708 | 0.000 | 0.398 | 3.275 | -2.876 | 0.000 | 0.211 | 1.354 | -1.143 | 0.000 |
| 6-12 months |  |  |  |  |  |  |  |  |  |  |  |  |
| ICU | 0.027 | 0.059 | -0.032 | 0.082 | 0.021 | 0.087 | -0.066 | 0.042 | 0.030 | 0.046 | -0.015 | 0.489 |
| HDU | 0.015 | 0.028 | -0.014 | 0.200 | 0.024 | 0.030 | -0.006 | 0.794 | 0.010 | 0.027 | -0.017 | 0.122 |
| GEN | 0.842 | 1.353 | -0.511 | 0.001 | 1.036 | 1.737 | -0.701 | 0.024 | 0.749 | 1.168 | -0.419 | 0.011 |
| IP | 0.028 | 0.048 | -0.020 | 0.147 | 0.048 | 0.048 | 0.000 | 0.996 | 0.019 | 0.047 | -0.029 | 0.042 |
| OP | 4.071 | 4.566 | -0.495 | 0.000 | 4.165 | 4.759 | -0.594 | 0.001 | 4.027 | 4.473 | -0.447 | 0.000 |
| DC | 0.399 | 0.629 | -0.230 | 0.000 | 0.477 | 0.888 | -0.411 | 0.000 | 0.362 | 0.504 | -0.142 | 0.004 |
| >12 months |  |  |  |  |  |  |  |  |  |  |  |  |
| ICU | 0.050 | 0.053 | -0.003 | 0.874 | 0.057 | 0.065 | -0.008 | 0.801 | 0.047 | 0.047 | -0.000 | 0.993 |
| HDU | 0.063 | 0.081 | -0.018 | 0.520 | 0.053 | 0.062 | -0.009 | 0.775 | 0.068 | 0.091 | -0.023 | 0.561 |
| GEN | 2.317 | 2.474 | -0.158 | 0.467 | 2.466 | 2.065 | 0.402 | 0.266 | 2.245 | 2.672 | -0.427 | 0.113 |
| IP | 0.133 | 0.110 | 0.023 | 0.537 | 0.147 | 0.094 | 0.053 | 0.374 | 0.126 | 0.117 | 0.008 | 0.859 |
| OP | 8.623 | 8.755 | -0.132 | 0.615 | 8.747 | 8.380 | 0.367 | 0.442 | 8.563 | 8.936 | -0.373 | 0.236 |
| DC | 1.319 | 1.441 | -0.121 | 0.330 | 1.311 | 1.498 | -0.188 | 0.397 | 1.323 | 1.413 | -0.089 | 0.553 |
| *N* | 3,035 | 3,030 | 6065 |  | 984 | 987 | 1,971 |  | 2,051 | 2,043 | 4,094 |  |
| Note. ICU- intensive care unit, HDU – high dependency unit, GEN – general medicine/acute, IP – inpatient visit, OP – outpatient visit, DC – day case | | | | | | | | | | | | |

Supplementary 4. Subgroup analysis

| Table S6: Subgroup cost-effectiveness results | | | | | | | | | |
| --- | --- | --- | --- | --- | --- | --- | --- | --- | --- |
|  | Costs (£/patient) | | LE | | QALYs | | NMB (£30K/QALY) | | Prob CE |
| Intervention strategies | Mean | 95% CI | Mean | [95% CI] | Mean | [95% CI] | Mean | [95% CI] |  |
| Treatment: **CAPOX** |  |  |  |  |  |  |  |  |  |
| *3Months* | 17,650 | [16,668; 18,919] | 6.90 | [6.75; 7.04] | 5.34 | [5.17; 5.48] | 142,500 | [136,469; 148,531] | 0.999 |
| *6Months* | 21,503 | [20,389; 22,723] | 6.83 | [6.67; 7.99] | 5.16 | [5.00; 5.32] | 133,253 | [128,206; 138,300] | 0.001 |
| *Incremental* | -3,853 | [-5,487; -2,030] | 0.07 | [-.13; .30] | .19 | [-.001; .38] | 9,247 | [4,178; 14,315] | (3M dominates) |
| **FOLFOX** |  |  |  |  |  |  |  |  |  |
| *3Months* | 19,641 | [18,477; 20,774] | 6.83 | [6.57; 7.03] | 5.21 | [4.99; 5.39] | 136,679 | [128,249; 145,108] | 0.772 |
| *6Months* | 26,483 | [24,659; 28,292] | 7.05 | [6.87; 7.23] | 5.33 | [5.15; 5.51] | 133,449 | [127,107; 139,791] | 0.228 |
| *Incremental* | -6,841 | [-9,040; -4,756] | -.22 | [-.52; .03] | -.12 | [-.38; .13] | 3,229 | [-2,494; 8,953] |  |
| Risk: **High risk** |  |  |  |  |  |  |  |  |  |
| *3Months* | 19,057 | [17,888; 20,339] | 6.31 | [6.11; 6.52] | 4.84 | [4.46; 5.03] | 126,450 | [118,973; 133,927] | 0.997 |
| *6Months* | 24,815 | [23,481; 26,362] | 6.36 | [6.15; 6.54] | 4.71 | [4.53; 4.87] | 116,477 | [110,700; 122,254] | 0.003 |
| *Incremental* | -5,758 | [-7,686; -3,924] | -0.05 | [-.32; .24] | .13 | [-.08; .37] | 9,972 | [4,664; 15,281] | (3M dominates) |
| **Low risk** |  |  |  |  |  |  |  |  |  |
| *3Months* | 17,671 | [16,596; 18,925] | 7.39 | [7.25; 7.52] | 5.72 | [5.58; 5.85] | 153,997 | [147,785; 160,208] | 0.922 |
| *6Months* | 21,422 | [20,305; 22,573] | 7.41 | [7.28; 7.53] | 5.71 | [5.55; 5.85] | 149,816 | [145,425; 154,206] | 0.078 |
| *Incremental* | -3,751 | [-5,402; -2,153] | -.02 | [-.20; .17] | .01 | [-.17; .21] | 4,180 | [-429; 8,791] | (3M dominates) |
| Gender: **Female** |  |  |  |  |  |  |  |  |  |
| *3Months* | 17,741 | [16408; 18934] | 6.92 | [6.74; 7.08] | 5.30 | [5.13; 5.47] | 141,617 | [133,821;149,413] | 0.997 |
| *6Months* | 22,228 | [20716; 23936] | 6.78 | [6.59; 6.94] | 5.10 | [4.92; 5.27] | 130,893 | [125,233; 136,553] | 0.003 |
| *Incremental* | -4,487 | [-6518; -2637] | 0.14 | [-.11; .39] | .20 | [-.03; .44] | 10,723 | [4,880; 16,567] | (3M dominates) |
| **Male** |  |  |  |  |  |  |  |  |  |
| *3Months* | 18,857 | [17,737; 20,098] | 6.83 | [6.63; 7.03] | 5.28 | [5.11; 5.43] | 139,377 | [132,810.1; 145,944] | 0.912 |
| *6Months* | 23,695 | [22,531; 24,930] | 6.98 | [6.82; 7.13] | 5.29 | [5.15; 5.83] | 135,055 | [130,064; 140,046] | 0.088 |
| *Incremental* | -4,837 | [-6,613; -3,011] | -.15 | [-.40; .09] | -.02 | [-.22; .18] | 4,321 | [-351; 8,994] |  |
| Age: $\boldsymbol{\geq}$**65** |  |  |  |  |  |  |  |  |  |
| *3Months* | 19,364 | [18,101; 20,831] | 6.73 | [6.57; 6.89] | 5.24 | [5.10; 5.39] | 137,918 | [131,146; 144,689] | 0.985 |
| *6Months* | 23,839 | [21,981; 24,917] | 6.72 | [6.52; 6.87] | 5.13 | [4.98; 5.27] | 130,294 | [125,355; 135,234] | 0.015 |
| *Incremental* | -4,474 | [-5,994; -2,090] | 0.01 | [-.22; .27] | .11 | [-.09; .32] | 7,623 | [2,789; 12,457] | (3M dominates) |
| **<65** |  |  |  |  |  |  |  |  |  |
| *3Months* | 17,572 | [16,431; 18,672] | 7.01 | [6.83; 7.17] | 5.35 | [5.18; 5.50] | 142,872 | [135,713; 150,032] | 0.977 |
| *6Months* | 23,066 | [21,838; 24,435] | 7.09 | [6.94; 7.24] | 5.29 | [5.12; 5.46] | 135,670 | [130,541; 140,799] | 0.023 |
| *Incremental* | -5,493 | [-7,297; -3,870] | -.08 | [-.32; .14] | .05 | [-.17; .28] | 7,202 | [1,609; 12,795] | (3M dominates) |
| Note 1. Health utilities conditional on survival considered.  Note 2. KMSA estimator and partitioned survival analysis used for costs and QALYs, respectively.  Note 3. CIs are computed using bootstrap sampling.  Note 4. Probability of cost-effectiveness calculated using 1000 bootstrap replications. | | | | | | | | | |

| Table S7. Overall survival time by health state (recurrence, DFS and ToT) and by arm. Subgroups | | | | | | | | |
| --- | --- | --- | --- | --- | --- | --- | --- | --- |
| By planned treatment | FOLFOX | | | | CAPOX | | | |
|  | 3M | 6M | Incremental | | 3M | 6M | Incremental | |
|  | Mean | Mean | Mean | P-value | Mean | Mean | Mean | P-value |
| Recurrence | .78 | .67 | .11 | 0.336 | .71 | .82 | -.10 | 0.336 |
| DFS | 5.81 | 5.96 | -.14 | 0.360 | 5.98 | 5.63 | .35 | 0.004 |
| ToT | .22 | .42 | .20 | <0.001 | .20 | .37 | -.17 | <0.001 |
| TOTAL (OS) | 6.82 | 7.05 | -.22 | 0.128 | 6.90 | 6.83 | .071 | 0.508 |
| *N* | 984 | 987 | 1,971 |  | 2,051 | 2,043 | 4,094 |  |
| By risk | High risk | | | | Low risk | | | |
|  | 3M | 6M | Incremental | | 3M | 6M | Incremental | |
|  | Mean | Mean | Mean | P-value | Mean | Mean | Mean | P-value |
| Recurrence | .95 | .86 | .08 | 0.434 | .55 | .71 | -.16 | 0.158 |
| DFS | 5.14 | 5.10 | .04 | 0.739 | 6.62 | 6.30 | .32 | 0.016 |
| ToT | .21 | .40 | -.18 | <0.001 | .21 | .39 | -.18 | <0.001 |
| TOTAL (OS) | 6.31 | 6.36 | -.048 | 0.736 | 7.39 | 7.42 | -.023 | 0.806 |
| *N* | 1,424 | 1,415 | 2,839 |  | 1,611 | 1,615 | 3,226 |  |
| By gender | Female | | | | Male | | | |
|  | 3M | 6M | Incremental | | 3M | 6M | Incremental | |
|  | Mean | Mean | Mean | P-value | Mean | Mean | Mean | P-value |
| Recurrence | 0.60 | 0.67 | -0.07 | 0.576 | .82 | .84 | -.02 | 0.817 |
| DFS | 6.10 | 5.73 | 0.37 | 0.006 | 5.80 | 5.74 | .053 | 0.690 |
| ToT | 0.21 | 0.38 | -0.16 | <0.001 | .21 | .40 | -.19 | <0.001 |
| TOTAL (OS) | 6.92 | 6.78 | 0.14 | 0.279 | 6.82 | 6.98 | -.16 | 0.195 |
| *N* | 1,199 | 1,194 | 2,393 |  | 1,836 | 1,836 | 3,672 |  |
| By age | $\geq$65 | | | | <65 | | | |
|  | 3M | 6M | Incremental | | 3M | 6M | Incremental | |
|  | Mean | Mean | Mean | P-value | Mean | Mean | Mean | P-value |
| Recurrence | 0.80 | 0.65 | 0.15 | 0.287 | .69 | .89 | -.20 | 0.042 |
| DFS | 5.72 | 5.69 | 0.03 | 0.838 | 6.10 | 5.78 | .31 | 0.013 |
| ToT | 0.21 | 0.38 | -0.16 | <0.001 | .21 | .41 | -.20 | <0.001 |
| TOTAL (OS) | 6.73 | 6.72 | 0.02 | 0.900 | 7.01 | 7.09 | -.077 | 0.518 |
| *N* | 1,525 | 1,540 | 3,065 |  | 1,510 | 1,490 | 3,000 |  |
| Note 1. Kaplan-Meier estimates used for computation of expected time in each health state.  Note 2. Survival time estimated up to 8 years post randomisation. | | | | | | | | |
